# Toward an effective delivery system of a microbial sink of the uremic toxin, p-cresol; an *in vitro* study with *Thauera aminoaromatica* S2

**DOI:** 10.3389/fmicb.2025.1577556

**Published:** 2025-05-21

**Authors:** Prakit Saingam, Rosita Rasyid, Britt Abrahamson, Thomas J. Lie, Bruce J. Godfrey, Jonathan Himmelfarb, Mari K. H. Winkler

**Affiliations:** ^1^Department of Civil and Environmental Engineering, University of Washington, Seattle, WA, United States; ^2^Department of Microbiology, University of Washington, Seattle, WA, United States; ^3^Kidney Research Institute, University of Washington, Seattle, WA, United States; ^4^Division of Nephrology, The Mount Sinai Center for Kidney Disease Innovation, Icahn School of Medicine at Mount Sinai, New York, NY, United States

**Keywords:** uremic toxin, hydrogel encapsulation, oral delivery method, p-cresol degrading microorganism, intestinal p-cresol removal

## Abstract

Individuals with chronic kidney disease (CKD) suffer from uremia, a condition characterized by the accumulation of uremic toxin in the blood. The aromatic uremic toxin p-cresol, a byproduct of tyrosine fermentation in the gut, binds to plasma albumin and cannot be removed with dialysis. However, the ingestion of densified p-cresol degrading microorganisms encapsulated in protective hydrogel beads could provide a therapeutic benefit by removing p-cresol from the colon. In this study the p-cresol degradation capacity of a known anaerobic, p-cresol degrading microorganism, *T. aminoaromatica* S2, encapsulated in polyvinyl alcohol and sodium alginate (PVA/SA) hydrogels was evaluated as a potential oral delivery method for intestinal p-cresol removal. Planktonic degradation was induced through prior p-cresol exposure, yielding a 100% removal efficiency at a rate of 92 nmol (Log CFU)^*-*1^ h^*-*1^ when exposed to 1.2 mM of p-cresol at 37°C. Increasing p-cresol concentrations inhibited p-cresol degradation. Hydrogel encapsulation of the bacteria supported high cell density packaging at 2.5 Log CFU (mL hydrogel)^*-*1^ and high activity right after hydrogel production, and more rapid activity than the planktonic cells, providing a powerful p-cresol-consuming microbial sink. Our experimental design mimicked distal colon conditions with an initial p-cresol level of 0.60 mM and at a pH 7 where the p-cresol degradation capacity of encapsulated culture was 2.3 × 10^3^ nmol (Log CFU)^*-*1^ h^*-*1^. The encapsulation of 10-fold increased cell concentrations resulted in more than 2-fold increased degradation rates. With the cell densification, the estimated daily hydrogel intake could be reduced from 134 mL to 58 mL to match daily exposure, thereby achieving mass balance. The effective removal rates were due to well distribution of bacteria cells within the hydrogels. The hydrogels with p-cresol pre-induced biomass showed immediate p-cresol removal even at p-cresol higher than 1.0 mM concentration. The current study demonstrated the potential application of encapsulated *T. aminoaromatica* S2 for the removal of colon p-cresol hence offloading the kidney from processing protein-bound uremic toxins. Further research in hydrogel design could yield efficient removal as well as cell encapsulation.

## Introduction

Chronic kidney disease (CKD) is a public health problem affecting 843.6 million people worldwide, 35.5 million people in the US according to the Centers for Disease Control and Prevention (Centers for Disease Control and Prevention, 2023; Jager et al., 2019). Protein-bound uremic toxins (PBUTs) such as p-cresol sulfate and indoxyl sulfate, all of which originate in the gut from dietary intake and/or microbial metabolism, are well-known constituents to cause toxicity in CKD patients (Harrison et al., 2021). Phenolics such as p-cresol and indole originate in the colon from the fermentation of the amino acids tyrosine and tryptophan, respectively, before being sulfonated to their PBUT forms, p-cresyl sulfate and indoxyl sulfate in the liver (Gryp et al., 2017; Niwa, 2010).

**Figure 1:**
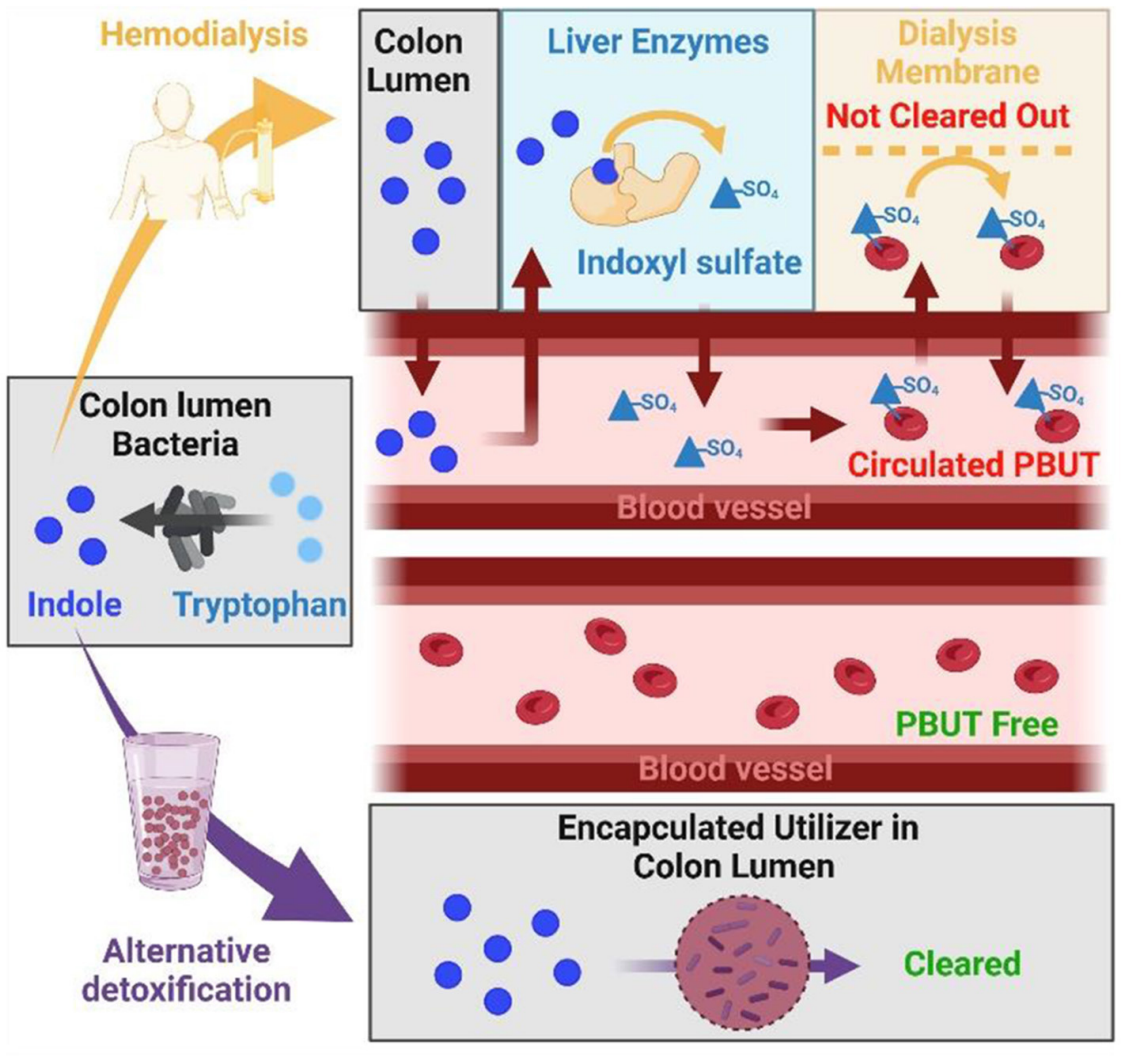
PBUT in CKD patient not cleared with dialysis while alternative detoxification removes PBUT from gut, preventing it from entering the blood. From left to right: Amino acids as tryptophan are converted into indole by colon bacteria. In patients receiving dialysis (upper panel), indole diffuses into the blood vessels and gets sulfonated in liver to indoxyl sulfate which shows strong affinity to albumin in the blood, which limits dialysis efficiency. Alternatively, bacteria encapsulated in hydrogels (bottom panel) could consume indole from the colon, which would prevent generation of indoxyl sulfate and result in PBUT-free blood circulation. The figure was created with BioRender.com.

Dialysis is the leading therapy when kidneys lose their capacity to remove toxins from the blood (Fletcher et al., 2022), however it fails to fully match the function of healthy kidneys. For example, small solutes (represented by urea and creatinine) are efficiently removed from the blood with a dialysis treatment however, aromatic PBUTs such as indoxyl sulfate and p-cresyl sulfate bind to plasma albumin and remain on the blood side of the dialysis membranes with at best modest dialytic clearance (Figure 1; Dhondt et al., 2000), and therefore alternative detoxification strategies are a critical unmet need.

The impact of the human gut microbiome on human health is extensive (Mills et al., 2019; Sommer and Bäckhed, 2013; Walter and Ley, 2011) and there is a growing appreciation for the intersection of diet and the gut microbiota as a driver of host wellbeing (Meijers et al., 2019; Mulle et al., 2013; Pluznick, 2020) including kidney health. Altering the production or removal of these toxins from the gut by chemical binders or by targeted changes to the human gut microbiome has been proposed. This concept is derived from studies showing that an imbalanced intestinal microbial community composition with quantitative and qualitative alterations in microbial abundances and variations in metabolic activities of the gut microbiota was observed during CKD (Hida et al., 1996; Vaziri et al., 2013). This offers promise to remove PBUTs from the colon before they enter the blood stream where damaged kidneys fail to remove them causing impaired health.

Recent studies demonstrated the removal of PBUT substrate p-cresol and indole by probiotic bacteria (Candeliere et al., 2022; Stuivenberg et al., 2022). According to the *in-vitro* tests, five yogurt-origin *Bifidobacterium* strains indicated approximately close to 50% removal of 0.002–2 mM p-cresol during their 24 h fermentative growth in MRS media at 37°C (Stuivenberg et al., 2022). In the other study, washed cells of *Bifidobacterium* and *Lactobacillaceae* strains indicated 0–21.4% removal of 1 mM p-cresol during anaerobic incubation in phosphate buffer for 48 h (Candeliere et al., 2022). Although these lactic acid bacteria demonstrated potential capability of microbial removal of PBUTs using gut-targeted approaches, their implementation as therapeutic product is still subject to further investigations. Some of these bacteria do not possess canonical genes for p-cresol and indole catabolic enzymes and the removal mechanisms are currently unknown.

Degradation of PBUT substrates coupled to denitrification can be leveraged as a promising therapeutic solution for preventing uremic toxin accumulation and nitrogen oxides (NO_x_) toxicity (Chen et al., 2024). Previously, p-cresol and indole utilization as the sole carbon source was reported for denitrifying bacteria, *Thauera aminoaromatica* S2 (Mechichi et al., 2002). Denitrification is a stepwise process, consisting of nitrate (${\text{NO}}_{3}^{-}$) reduction to nitrite (${\text{NO}}_{2}^{-}$) and then to nitric oxide (NO), and nitrous oxide (N_2_O) to finally yield harmless nitrogen gas (N_2_) via four enzyme reductases. The human colon environment is enriched with nitrogen oxide species (NO_x_ where x represents the different number of oxygen molecules), which are potential substrates for denitrification (Sparacino-Watkins et al., 2014). ${\text{NO}}_{3}^{-}$ and ${\text{NO}}_{2}^{-}$ can be in food products and have been mentioned to be residing and available within the human gut based on traditional evidence of NO_3_/NO_2_ in the ileum fluid and the feces (Bartholomew and Hill, 1984). Moreover, the *in-vitro* studies pointed out the potential of several gut commensals in reducing NOx via denitrification (Tiso and Schechter, 2015). The epithelial cells also play a role in making NO available (Lundberg and Weitzberg, 2013). Denitrifiers, which possess enzymes degrading p-cresol and indole as well as their intermediates would oxidize the aromatic substrates completely to CO_2_ while coupling it to denitrification. In addition to *T. aminoaromatica* S2, diverse denitrifiers (Meza-Escalante et al., 2015; Levine et al., 2006; Szogi et al., 2018) were shown to degrade p-cresol and indole. Implementation of a gel encapsulated PBUT degrading denitrifier targeted to the colon environment could provide not only reduction of PBUTs but also control NO_x_ levels, which may compound the negative health effects of CKD (Vahid et al., 2012).

*T. aminoaromatica* S2 has high potential to be used for gut p-cresol removal. The NO_x_ can be supplied through dietary intake and endogenous synthesis (Lundberg and Weitzberg, 2013). Complete p-cresol oxidation can be coupled to denitrification with various nitrogen oxides as electron acceptors to drive their metabolism. One mole of p-cresol stoichiometrically reacted with two moles of ${\text{NO}}_{3}^{-}$ to produce one mole of p-OH benzoic acid and two moles of ${\text{NO}}_{2}^{-}$, which can both be further picked up and metabolized by the gut microbiome. As such, denitrifying bacteria further activate p-cresol hydroxybenzoate to p-hydroxybenzoyl-coenzyme A (CoA) by ligase (Biegert et al., 1993; Gibson et al., 1994). However, it would be best if degradation pathways are not truncated across different microbes but can be metabolized by one strain and a good example is *T. aminoaromatica* S2 as it possesses enzymes for complete denitrification from ${\text{NO}}_{3}^{-}$ to N_2_ (with ${\text{NO}}_{2}^{-}$ as intermediate product; Liu et al., 2013) while also able to metabolize p-cresol making it an excellent candidate to study the PBUT removal.

Encapsulation of microorganisms in hydrogel vehicles has contributed to successful applications of environmental monitoring (Tang et al., 2021), waste treatment (Li et al., 2023), disease diagnosis (Lim et al., 2022), and therapeutic application (Li et al., 2022). Delivery of suspended microbial cells to the colon would be influenced by limitations to concentrate cells as well as by a washout effect due to the 20–40 h colon passage time limit (Degen and Phillips, 1996). In wastewater treatment process, encapsulation maintains high densities of slow-growing nitrifiers for effective removal of ammonia, preventing washout (Minh et al., 2021). Encapsulation also provides the advantage of concentrating microbial biomass, thus increasing the catabolic activity without increasing the required volume of the therapeutic solution. This is a sensitive issue as kidney patients strictly need to limit their fluid intake (Wagner et al., 2022).

In nitrifying sludge, it was demonstrated that an approximately six-fold increase in biomass of nitrifying sludge resulted in a more than 10-fold increase of oxygen uptake rate and ammonia oxidation activity (Minh et al., 2021). Colon passage of bacteria-immobilized hydrogel was previously investigated for removal of nitrogenous waste of urea and creatinine using bacteria isolates from the human gut (Zheng et al., 2020). The bacteria were encapsulated in sodium alginate-based hydrogels, resulting in 50%−75% removal of urea and creatinine during 12-h incubation in the *in-vitro* tests. Personalized therapeutic practice could be achieved with use of customized microbial combinations encapsulated in hydrogels targeted to the colon for an effective delivery of microbes.

In this study, we demonstrated the use of *T. aminoaromatica* S2 in a hydrogel vehicle to remove PBUT substrate p-cresol in the gut-like condition. The study aimed to investigate (1) p-cresol removal by planktonic *T. aminoaromatica* S2 at 37°C at different p-cresol concentrations, (2) p-cresol removal by encapsulated bacteria, (3) whether the rate could be enhanced by encapsulating higher numbers of bacterial cells within hydrogels, and (4) p-cresol removal persistency at variable concentrations of p-cresol. This proof-of-concept study provided evidence that using microbial capability in the gut may be effective to remove PBUTs from a patient's body and that this approach could be developed into a therapeutic solution for gut centered kidney disease treatment.

## Method

### Bacterial strain

*T. aminoaromatica* S2 (DSMZ 14742) was obtained from DSMZ bacteria collection (Braunschweig, Germany). Subcultures were prepared in a bacteria cultivation media with acetate as a carbon source and ${\text{NO}}_{3}^{-}$ as an electron acceptor under anaerobic cultivation at 30°C. Culture purity was confirmed and a glycerol stock (25% v/v) was prepared and stored in −80°C.

### Bacteria cultivation media

*T. aminoaromatica* S2 was cultivated in basal media supplemented with vitamin solution, mineral solution, NaH_2_PO_4_, NaHCO_3_, Na_2_CO_3_, acetate, and ${\text{NO}}_{3}^{-}$. The basal media, vitamin solution, mineral solution, acetate, and ${\text{NO}}_{3}^{-}$ were prepared as anaerobic stock solutions. The basal media contained 200 mg/L NH_4_Cl, 100 mg/L KCl, 492.8 mg/L MgSO_4_·7H_2_O, 14.68 mg/L CaCl_2_·2H_2_O, and 10 mg/L NaCl. The vitamin solution consisted of 2 mg/L d-biotin, 2 mg/L folic acid, 10 mg/L pyridoxine HCl, 5 mg/L riboflavin, 5 mg/L thiamin, 5 mg/L nicotinic acid, 5 mg/L pantothenic acid, 0.1 mg/L vitamin B12, 5 mg/L p-aminobenzoic Acid, and 5 mg/L alpha lipoic Acid. The mineral solution consisted of 1,500 mg/L NTA disodium salt, 3,000 mg/L MgSO_4_·7H_2_O, 500 mg/L MnSO_4_·H_2_O, 1,000 mg/L NaCl, 100 mg/L FeSO_4_·7H_2_O, 100 mg/L CaCl_2_·2H_2_O, 100 mg/L CoCl_2_·6H_2_O, 130 mg/L ZnCl, 10 mg/L CuSO_4_·5H_2_O, 10 mg/L AlK(SO_4_)_2_·12H_2_O, 10 mg/L boric acid, 25 mg/L Na_2_MoO_4_·2H_2_O, 24 mg/L NiCl_2_·6H_2_O, 25 mg/L Na_2_WO_4_·2H_2_O, and 20 mg/L Na_2_SeO_3_. Stocks of NaHCO_3_, NaH_2_PO_4_, Na_2_CO_3_, acetate, and ${\text{NO}}_{3}^{-}$ were 90 g/L, 80 g/L, 50 g/L, 1.7 M, and 500 mM, respectively. All stock solutions were sterilized using 0.22 μm membrane filter and gassed with either N_2_ or 80% N_2_:20% CO_2._ After the autoclave sterilization, oxygen in the headspace of the basal media was then removed with 80% N_2_:20% CO_2_ flushing. While the headspace was being flushed, 10 mL vitamin solution, 10 mL mineral solution, 7.5 mL NaH_2_PO_4_ and 28 mL NaHCO_3_ were added into the 955 mL anaerobic basal media, and the media was aliquoted into autoclave sterilized Balch-type tubes or serum vials pressurized with 80% N_2_:20% CO_2._ Prior to the cultivation, the media aliquot was supplemented with 30 mM acetate, 25 mM ${\text{NO}}_{3}^{-}$, and 1.75 g/L Na_2_CO_3_. The pH of final media was 7.0.

### Bacteria biomass cultivation

The inoculum for biomass cultivation for each experiment was from a single colony revived aerobically on R2A media at 30°C for 48 h from the glycerol stock. A single colony was inoculated into a Balch-type tube of 10 mL media and cultivated at 30°C until the cell density reached 1 OD_600_. To culture more biomass, the 10 mL culture was used as the inoculum for the serum vial of 200 mL media (3% v/v) and the cultivation was repeated to reach 1 OD_600_. The addition of ${\text{NO}}_{3}^{-}$ was performed twice, 10 and 15 mM to prevent the ${\text{NO}}_{2}^{-}$ toxicity.

### Bacteria p-cresol induction and encapsulation of cells

The biomass for the p-cresol removal tests by both planktonic and encapsulated cells was induced with prior p-cresol exposure. P-cresol degradation activity was initially induced by centrifuging the acetate grown *T aminoaromatica* S2 (1 OD_600_) at 1,000 × g for 30 min, washing the cell pellet twice with media without carbon sources and electron acceptors, and resuspending the cell pellet in the similar volume of media supplemented with 0.6 mM p-cresol and 10 mM ${\text{NO}}_{3}^{-}$. The p-cresol-fed cultures were incubated at 30°C.

After the more than 50% removal of p-cresol was confirmed through analysis using LC system, a sample was collected for bacteria cell quantification. The culture was divided into two portions, and one was heated at 92°C for 30 min to be used as abiotic control (Wang et al., 2012). The cell pellet of the two portions was collected and washed twice with the basal media. The washed cells were directly transferred to the p-cresol removal test as planktonic cells or encapsulated as hydrogels. Hydrogel encapsulated cells were prepared by resuspending the cell pellet in 6% w/v polyvinyl alcohol (PVA; Mw = 89,000–98,000, 99+% hydrolyzed, Sigma-Aldrich, St. Louis, MO) and 2% sodium alginate (SA; Cape Crystal Brands, Summit, NJ) solution with ~2–3 Log CFU (mL hydrogel)^*-*1^ cell encapsulation density. The cell resuspension was extruded through a 21G sterilized syringe needle into 4% (w/v) CaCl_2_. The hydrogels were stirred for 1 h in the anaerobic atmosphere prior to being processed for the p-cresol removal test. Most of the preparation of bacteria cells for p-cresol induction and hydrogel encapsulation was performed in anaerobic conditions using Hungate techniques (Hungate, 1969) or using the Coy(TM) anaerobic chamber (85% N_2_: 10% CO_2_: 5% H_2_ atmosphere). All the solutions and glassware used for encapsulation were autoclaved.

### P-cresol removal by planktonic and encapsulated cells

The planktonic cells and encapsulated hydrogels were rinsed and resuspended in the modified media. Use of modified media was mainly to prevent ionic effects on the hydrogel stability. In the modified media, the NaHCO_3_ and Na_2_CO_3_ were excluded from the basal media mentioned above. The modified media was supplemented with vitamin, mineral solutions, and with 10 mM ${\text{NO}}_{3}^{-}$. The pH was adjusted to 7.0 with 1 M NaOH and the headspace was filled with N_2_ gas. The supplemented p-cresol was at 0.6–5.2 mM based on the concentration range detected in gut tissues, feces, and *in-vitro* study of gut bacteria (Gryp et al., 2020; Smith and Macfarlane, 1996; Passmore et al., 2018; Saito et al., 2018). In each experiment, the non-heated cells and heated cells (abiotic control) were separated into three portions serving as triplicate measurements. Each replicate was incubated in shaking condition 133 rpm at 37°C. The media of 15 mL in a Balch-type tube and of 40 mL in a serum vial were prepared for the planktonic cell and the encapsulated cell test, respectively. The preparation of planktonic cells and encapsulated hydrogels for the p-cresol removal test was prepared using either Hungate techniques (Hungate, 1969) or the anaerobic chamber. Bulk liquids were collected for measurements of p-cresol, ${\text{NO}}_{3}^{-}$ and ${\text{NO}}_{2}^{-}$ levels, and bacteria cell quantification. Hydrogels were also collected for microscopic investigation and bacteria cell quantification. The wet weight of bacteria hydrogels being prepared for each replicate and being collected for analyses was also recorded for result normalization. The total extracted volume of bulk liquid and hydrogels were < 30% of the initial volume.

### Chemical analysis

The cultures were filtered through 0.2 μm nylon filter (VWR, Radnor, PA) for analysis of total nitrogen, ${\text{NO}}_{2}^{-}$, and p-cresol. Total nitrogen-N and ${\text{NO}}_{2}^{-}$-N were analyzed via spectrophotometric measurements using Gallery™ Automated Photometric Analyzer and reagents from the manufacturer (Thermo Scientific, Waltham, MA) following the manufacturer's protocol. The analyses were performed following standard methods (WEF, 2005). Concentrations of p-cresol and intermediates were quantified by 1260 Infinity II LC system (Agilent Technologies, CA) with Aminex HPX-87H column (Biorad laboratories, CA). The separation was achieved through mixed mobile phase of acetonitrile and 0.5 mM H_2_SO_4_ (2:8 volume ratio) with 0.6 mL/min flowrate at 30°C. The chromatogram was monitored at UV 210 nm. The p-cresol removal rates by planktonic culture were reported as nmol (Log CFU)^*-*1^ h^*-*1^. The p-cresol removal rates by encapsulated bacteria were reported as nmol (Log CFU)^*-*1^ h^*-*1^ or nmol (mL hydrogel)^*-*1^ h^*-*1^. The calculations were described in the Supplementary material S.2.1. Biological triplicates were calculated for arithmetic mean and standard deviations (SD).

### Hydrogel microscopy

The hydrogels were photographed and determined for diameter size using a zoom stereo microscope and Zen software. The hydrogels were also processed for cell fixing by storing in 2% PFA solution at 4°C until further processing. The fixed samples were embedded in the Neg-50 section medium (Richard-Allan Scientific, Kalamazoo, USA) at −20°C and cross-sectioned using a CryoStar NX50 cryotome (Thermo Scientific, Waltham, USA). The bacteria cells in the cross-sectioned slice were stained using SYBR dyes. The SYBR staining was performed using dilution of the commercial stock solution and incubated in the dark at 37°C for 30 min. The photography of both light microscopy images of the bead and green fluorescence images was performed using a Axioskop 2 mot plus fluorescence microscope (Carl Zeiss, Germany).

### Bacteria cell quantification

The cell numbers were quantified using the standard plate count method. The planktonic culture and bulk liquid samples were directly serial diluted in PBS buffer for plate counts. Two to three hydrogels were sampled and rinsed with sterile water. The hydrogels were further vortexed briefly in 1 mL of 20 mM sodium citrate in PBS buffer to release encapsulated cells (Jeong et al., 2023). The suspension was incubated at room temperature until complete dissolution. The dissolved hydrogel suspension was serial diluted for plate counts. For the plate counting, 50 μL of diluted culture was spread on R2A media, and the media was incubated aerobically at 30°C for 5 days, in which maximum growth of *T. aminoaromatica* was observed. Only plates showing countable number of colonies (30–300 CFU) were used to determine the cell quantity. The cell quantity was reported as Log CFU and the cell encapsulation density was reported as Log CFU (mL hydrogel)^*-*1^. Biological triplicates were calculated for arithmetic mean and standard deviations.

## Results

### P-cresol removal by planktonic *T. aminoaromatica* S2

**Figure 2:**
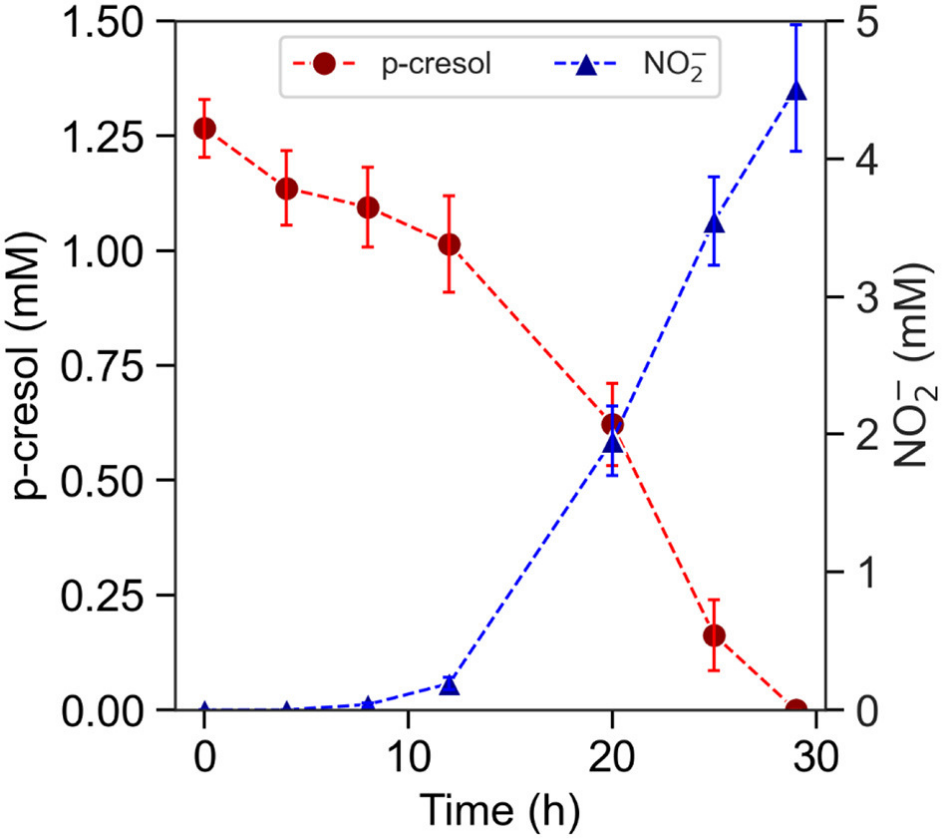
P-cresol removal by pre-induced planktonic culture of *T. aminoaromatica* S2 at 37°C. Measured concentrations of p-cresol and measured concentration of ${\text{NO}}_{2}^{-}$. The standard deviation is based on biological triplates.

P-cresol removal by planktonic *T. aminoaromatica* S2 was evaluated for p-cresol concentrations ranging between 1.2 and 5.2 mM, which reflects the concentrations detected in gut tissues, feces, and *in-vitro* study of gut bacteria (Gryp et al., 2020; Smith and Macfarlane, 1996; Passmore et al., 2018; Saito et al., 2018). The initial cell concentration was 8.0 (SD = 0.40) Log CFU. In the culture supplemented with 1.2 mM p-cresol and 10 mM ${\text{NO}}_{3}^{-}$, the removal of p-cresol was observed immediately in the first 4 h (Figure 2) and coincided with the accumulation of benzoic acid, a p-cresol degradation intermediate (Supplementary Figure S1). The denitrification by-product ${\text{NO}}_{2}^{-}$ was not detected until hour eight, followed with a brief lag-phase and accelerated rate since hour 12, demonstrated by the trend of ${\text{NO}}_{2}^{-}$. The p-cresol concentration of 1.2 mM was completely removed after 29 h. The level of produced ${\text{NO}}_{2}^{-}$ was 4.5 (SD = 0.46) mM at after 29 h. The absolute removal rate was 92 (SD = 4.6) nmol (Log CFU)^*-*1^ h^*-*1^.

**Figure 3:**
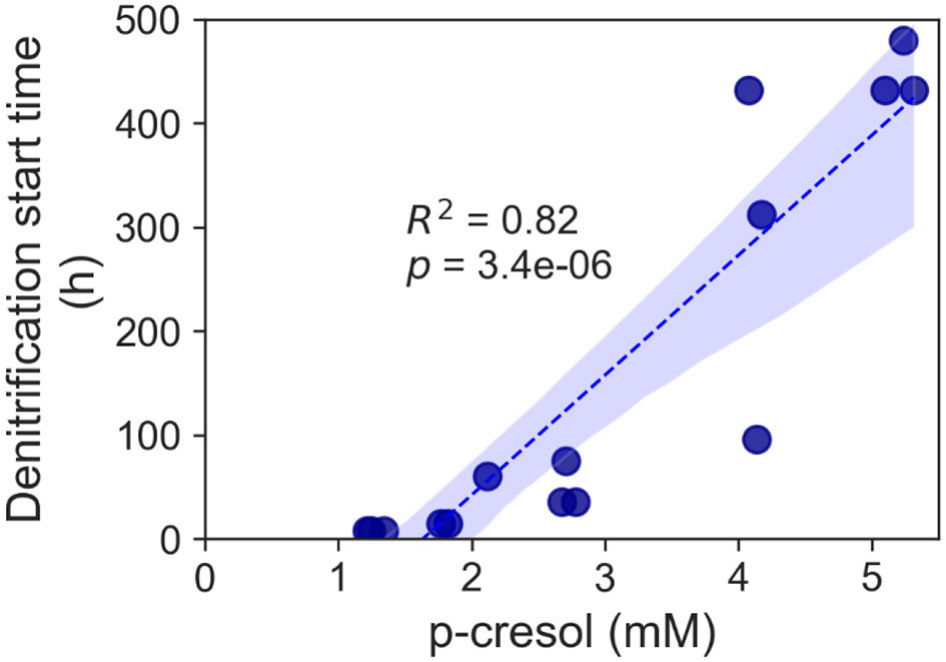
Increase of denitrification start time for the p-cresol removal of the *T. aminoaromatica* S2 planktonic culture. Each data point represented a biological replicate and p-cresol concentration range 2.1–5.3 mM.

Cultures supplemented with elevated p-cresol concentrations (2.1–5.3 mM) demonstrated a delayed onset of denitrification (Figure 3). The denitrification of the 2.1 mM did not start until hour 60 and not until hour 75 for the 2.7 mM. Furthermore, the conditions supplemented with higher than 4 mM concentrations required at least 2 weeks for denitrification. The time taken prior to start of p-cresol removal showed strong correlation to concentrations of p-cresol.

### P-cresol removal test of encapsulated *T. aminoaromatica* S2 and impact of biomass densification

**Figure 4:**
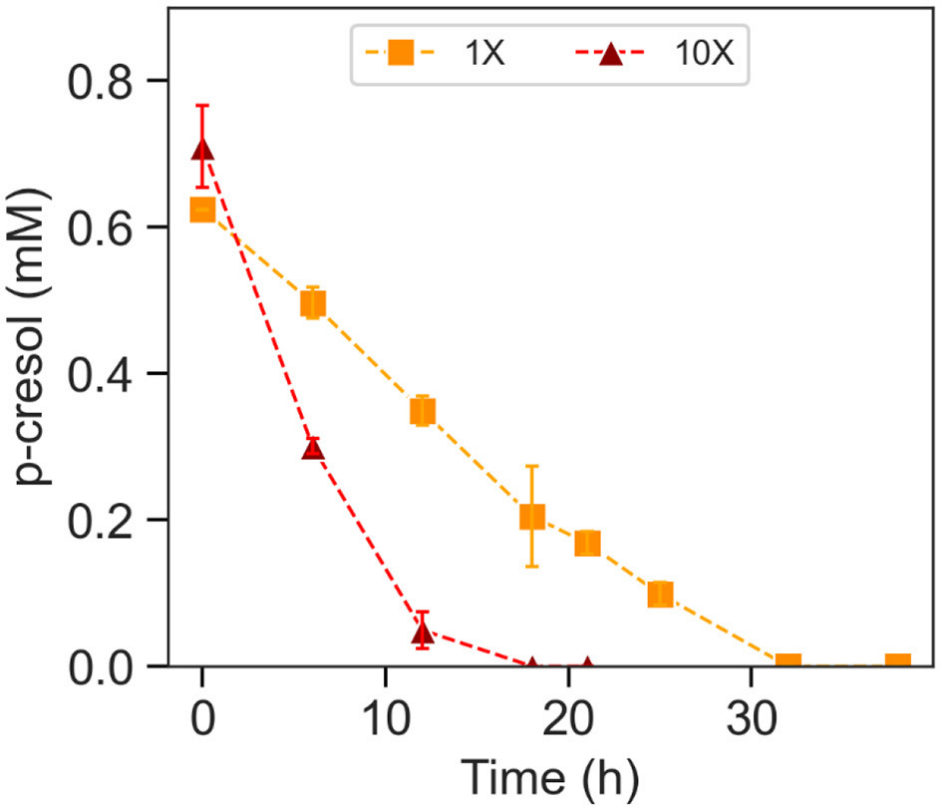
P-cresol removal during the second spiking of p-cresol by encapsulated *T. aminoaromatica* S2 at 37°C at pH 7. The p-cresol removal was demonstrated from two different biomass levels, 1× and 10×. The standard deviation is based on biological triplates.

Acetate-grown and p-cresol-exposed *T. aminoaromatica* S2 cells were encapsulated in PVA/SA hydrogels (Supplementary Figure S2) with two different cell densities in the hydrogel, regular (1×) and 10-fold concentrated (10×). The cells were observed to be well distributed across the hydrogel (Supplementary Figure S2). The plate count of cells extracted from the 10× beads showed 8.0 Log CFU (*n* = 1). 1× beads showed 7.9 (SD = 0.20; *n* = 3). The estimated p-cresol concentrations in the gut could be variable throughout the colon (Smith and Macfarlane, 1996). Therefore, there is a necessity to investigate the hydrogel versatility to repeated exposure to p-cresol, which was simulated by two additions of p-cresol. Both 1× and 10× hydrogels completely removed the first addition of ~0.60 mM within 34 h incubation at 37°C at pH 7, corresponding to the absolute removal rates of 2.3 × 10^3^ (SD = 89) nmol (Log CFU)^*-*1^ h^*-*1^ (Supplementary Figure S3 and Table 1). Both showed p-cresol benzoic acid intermediate of p-cresol and ${\text{NO}}_{2}^{-}$ denitrification product with the p-cresol removal pattern similar to the planktonic cells. The difference in removal rates between 1× and 10× was observed after the second addition of 0.60 mM p-cresol (Figure 4 and Table 1). The 10× densified hydrogel encapsulated cells removed 0.60 mM within 18 h, corresponding to the absolute removal rate of 4.9 × 10^3^ (SD = 3.8 × 10^2^) nmol (Log CFU)^*-*1^ h^*-*1^ (Figure 4 and Table 1). The 1× (not densified) hydrogel did not completely remove p-cresol until hour 32, corresponding to the absolute removal rate of 2.4 × 10^3^ (SD = 24) nmol (Log CFU)^*-*1^ h^*-*1^(Figure 4 and Table 1). The model fitting indicated that 10× removed p-cresol faster than 1× condition (*t*-test, *p*-value < 0.01). Abiotic control of both biomass levels did not indicate continued p-cresol removal after the initial minor drop of p-cresol and reduction of ${\text{NO}}_{3}^{-}$ (Supplementary Figure S4). Upon complete removal of the added p-cresol, the plate counting of 10× and 1× beads indicated 9.0 (SD = 0.080) and 7.9 (SD = 0.65) Log CFU, respectively. In the meantime, 0.23 (SD = 0.078)% and 0.18 (SD = 0.17)% of the total cells encapsulated in hydrogels were observed in the bulk liquid, indicating that cells escaped from the hydrogels or growth of culture in the media. In order to verify the p-cresol removal capability of encapsulated cells, a comparative test was performed by relating p-cresol removal rate obtained from encapsulated hydrogel with bulk liquid containing cells to the rate of the bulk liquid of cells alone (Supplementary Figure S5). Within 30 h, the hydrogel culture showed complete removal of 0.60 mM whereas the cells in bulk liquid alone did not show specific changes in p-cresol level, demonstrating removal activity predominantly contributed by cells encapsulated within the hydrogels.

**Table 1:** Absolute removal rate of p-cresol by encapsulated *T. aminoaromatica* S2 in neutral pH condition and at two different cell density level (10× and 1×).

**Cell density**	**nmol (Log CFU)**^*****-***1**^ **h**^*****-***1**^		**nmol p-cresol mL hydrogel**^*****-***1**^ **h**^*****-***1**^	
	**1st p-cresol addition**	**2nd p-cresol addition**	**1st p-cresol addition**	**2nd p-cresol addition**
10 ×	2.3 × 10^3^	4.9 × 10^3^	6.3 × 10^3^	1.3 × 10^4^
1 ×	2.3 × 10^3^	2.4 × 10^3^	5.8 × 10^3^	5.8 × 10^3^

### Effect of p-cresol concentration on removal with hydrogel encapsulated *T. aminoaromatica* S2

**Figure 5:**
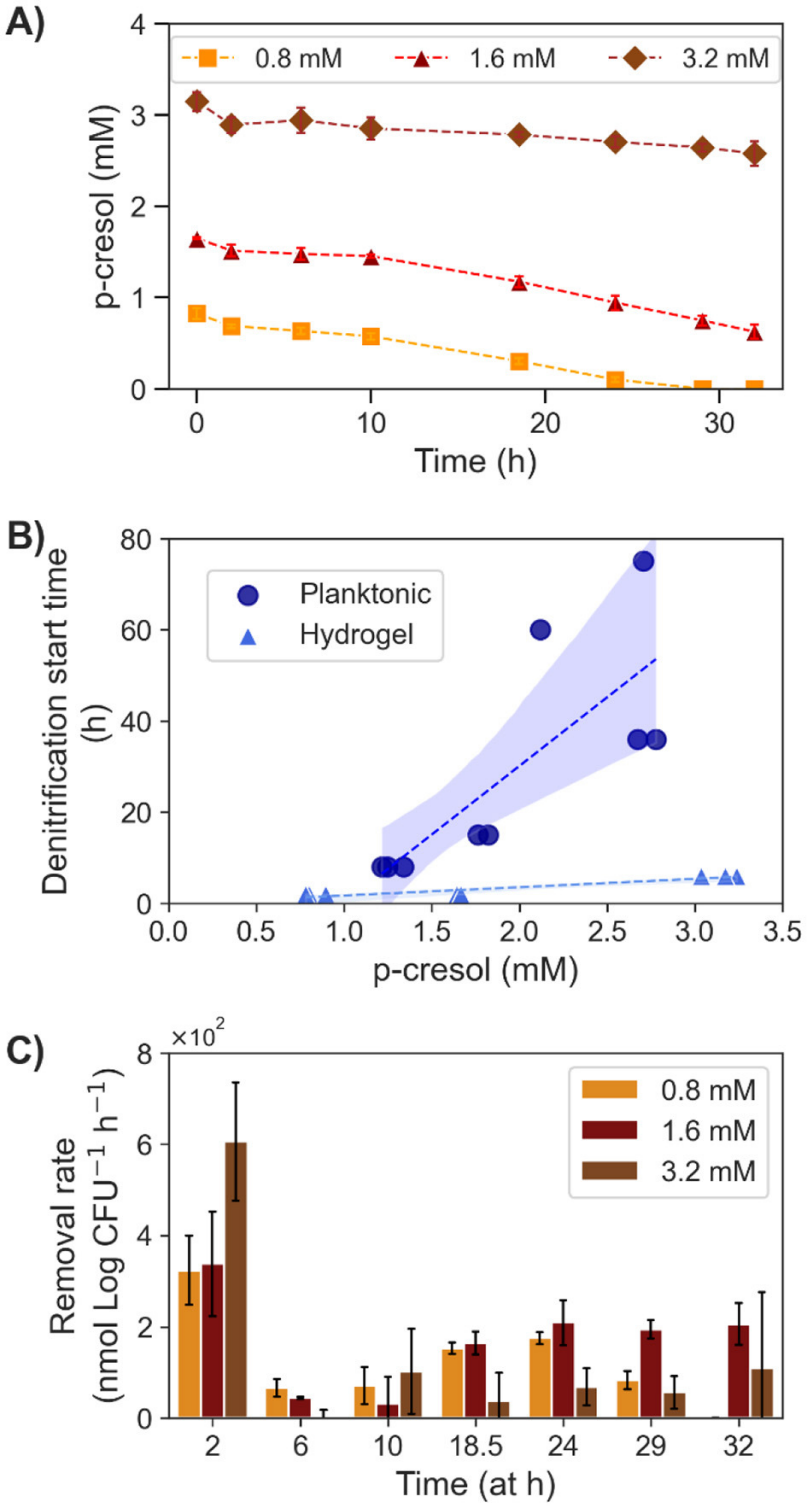
P-cresol removal by encapsulated *T. aminoaromatica* S2 at 37°C at three different initial concentrations (0.8, 1.6, and 3.2 mM). **(A)** Measured p-cresol concentration, **(B)** the observed denitrification start time compared with the planktonic culture, and **(C)** the calculated step wised removal rates. The values shown on the plot **(A, C)** were average with SD from three biological replicates. Each data point on the plot **(B)** represented a biological replicate.

The removal of p-cresol with hydrogel encapsulated *T. aminoaromatica* S2 was evaluated at three different p-cresol concentrations (0.80, 1.6, and 3.2 mM; Figure 5). The cell encapsulation density was 2.7 (SD = 0.19) Log CFU (mL hydrogel)^*-*1^. The initial cell amount in each concentration test was 8.3 (SD = 0.55) Log CFU. All three conditions demonstrated the initial drop of p-cresol like the planktonic cultures (Figure 5A). However, the time until the start of denitrification was closer among the biological replicates and across the tested concentrations (Figure 5B). Stepwise calculated removal rates revealed similar pattern of measured removal rates across three concentrations (Figure 5C). The hydrogel showed the initial spike of removal, a lag-phase, and the persistently increasing p-cresol removal. The maximum removal rates from the 0.80 and 1.6 mM initial concentration was 1.8 × 10^2^ (SD = 14) nmol (Log CFU)^*-*1^ h^*-*1^ and 2.1 × 10^2^ (SD = 50) nmol (Log CFU)^*-*1^ h^*-*1^. Whereas, the maximum for the 3.2 mM was 1.1 × 10^2^ (SD = 1.7 × 10^2^) nmol (Log CFU)^*-*1^ h^*-*1^. Toward hour 29 and 32, the removal rates of 0.80 mM decreased, which coincided with depletion of p-cresol. The conditions 0.80 and 1.6 mM showed higher absolute removal rates [1.2 × 10^2^ (SD = 9.1) and 1.6 × 10^2^ (SD = 18) nmol (Log CFU)^*-*1^ h^*-*1^] than the 3.2 mM condition [84 (SD = 6.7) nmol (Log CFU)^*-*1^ h^*-*1^]. In the meantime, the changes in cell quantity within hydrogels through the 32 h incubation showed consistent results as the conditions of 0.8 and 1.6 mM showed increase, in contrast to the decrease observed with 3.2 mM condition. The cell quantity was 8.9 (SD = 0.30) and 8.7 (SD = 0.62) Log CFU of condition 0.8 and 1.6 mM, respectively, whereas 3.2 mM showed less [7.3 (SD = 0.29) Log CFU]. During the experiments, a maximum of 13% cells were present in the bulk liquid. The comparative tests verified the degradation activity primarily due to the cells within the hydrogels.

## Discussion

The kidney's key function is to expel waste and toxin such as PBUT from the blood. As a result, impaired kidney function leads to elevated serum levels of PBUT among CKD patients, leading to renal effect progression, metabolic, and cardiovascular disease (Nataatmadja et al., 2017). In addition, dialysis is currently not effective to remove PBUTs (Dhondt et al., 2000) and an alternative treatment strategy for PBUT removal is currently missing. In order to prevent accumulation and negative health effects of PBUT among CKD patients, new removal strategies are needed. The bacterium *T. aminoaromatica* S2 is capable of completely catabolizing p-cresol (Carmona et al., 2009). In this study, *T. aminoaromatica* S2 cells were induced with exposure to p-cresol before evaluating the p-cresol removal rate at 37°C and anaerobic conditions simulating the human gut. In 1× culture, the cell encapsulation density was 2.5 (SD = 0.047) Log CFU (mL hydrogel)^*-*1^. Gut-generated p-cresol amount in CKD patients can be estimated from daily urine excretion of p-cresyl sulfate, which was reported to be 0.75 mmol per day (Chen et al., 2020, 2021). The highest removal rate observed was 2.4 × 10^3^ (SD = 24) nmol (Log CFU)^*-*1^ h^*-*1^, which would require consumption of 134 mL of hydrogel to remove a daily p-cresol generation (Supplementary material S.2.1). A major advantage of utilizing hydrogel entrapment as a delivery mode for the microbes is biomass densification. As shown in our study, a 10-fold increase in biomass in the hydrogel [10× culture, 2.82 Log CFU (mL hydrogel)^*-*1^] enhanced the removal rate up to 4.9 × 10^3^ (SD = 3.8 × 10^2^) nmol (Log CFU)^*-*1^ h^*-*1^, in which the hydrogel daily intake volume could be reduced to 58 mL (Supplementary material S.2.1). The treatment with microbial sink in the hydrogel allows biomass dosage modification and hence personalized precision medicine to minimize medication burden and positively impact life quality.

In this study, the 6% PVA 2% SA hydrogel was used for bacterial cell encapsulation. The SYBR staining indicated well distribution of the bacteria cell throughout the hydrogel. The hydrogel would cause diffusion limitation (Candry et al., 2022) of p-cresol, NO_x_, and other bacteria essentials, leading to a transient concentration gradient ranging from the highest at the periphery to lower concentrations toward the core of hydrogels. This could provide a protective mechanism and benefit the performance of the hydrogel p-cresol removal at high concentrations. The hydrogels demonstrated denitrification of p-cresol within 5 h vs. up to 75 h demonstrated from the planktonic cells. In the meantime, the presence of up to 9.71% free cells in the bulk liquid was observed. However, quantification demonstrated an increase in cells in the hydrogel, pointing to activity and growth of cells inside the hydrogels (Supplementary Table S1). In addition, the comparative tests demonstrated that the p-cresol removal activity was largely contributed by cells in the hydrogels.

Our hydrogel encapsulation also allows for delivery of combined microorganism types and chemical binders. In this study, we observed the presence of the intermediate of p-cresol catabolism, p-OH benzoic acid. While *T. aminoaromatica* S2 possess enzymes degrading intermediates further from p-OH benzoic acid, the accumulation of p-OH benzoic acid in this study might be due to unaccounted factors. P-OH benzoic acid was previously reported to slow down microbial metabolic rates (Manuja et al., 2013). However, at the same time benzoic acid is discussed to ameliorate cardiovascular problems (Juurlink et al., 2014). Future studies should therefore focus on, testing the impact of *T. aminoaromatica* S2 on cardiovascular improvements in an animal model and or consider the addition of p-OH benzoic-acid-degrading bacteria (Zhai et al., 2021) as part of a synergistic consortium within the hydrogel which could relieve the inhibition and help ensure the complete removal p-cresol hence avoiding a possible lag phase for p-cresol removal. Also, of importance, it is challenging to estimate p-cresol concentrations in the gut due to its dynamic processes of e.g., microbial biosynthesis and absorption through epithelial cells of gut lining (Meijers and Evenepoel, 2011). Samples of colon tissues from the proximal and distal part demonstrate p-cresol concentration in the range of 1 mM (Smith and Macfarlane, 1996) but concentrations may be higher. Densification of bacteria in the hydrogel could potentially lower higher concentrations quickly and also provide a protective milieu to the encapsulated microbes' hence providing ways to deal with variable p-cresol concentration in the gut. Hydrogel of p-cresol microbial sink could be developed as a therapeutic drink product, which provides a remedy to CKD conditions without causing major limitations and disruptions to patient's wellbeing.

While hydrogel encapsulated *T. aminoaromatica* S2 showed successful removal of p-cresol, several aspects including safety of the therapeutic use of environment-originated bacterial strains, hydrogel optimization, and realistic application require future investigations to achieve microbial sink product of gut PBUT removal. Like other live biotherapeutic products, the bacterial strain needs to be characterized to assess health risks such as virulence factors and antibiotic resistance (Dreher-Lesnick et al., 2017). PVA/SA hydrogel has been used in wide applications such as wound dressing (Kamoun et al., 2015), waste treatment (Candry et al., 2022), and energy production (Patel et al., 2020). The hydrogel is suitable for microbial application due to its non-toxic characteristics and robust against to dissolving during application with unknown ions or chelators. However, the PVA/SA hydrogel is subject to variations of porosity influencing diffusion and cell containment (Candry et al., 2022). While optimal condition for *T. aminoaromatica* S2 is at pH 7.5–8.6 and 28°C (Mechichi et al., 2002), further hydrogel optimization would help protect the bacteria through the gastrointestinal passage and improve the cell retention. For instance, a pH-responsive hydrogel as the alginate core-shell particles offered effective protection during the gut transit and achieved microbial diagnosis of gut inflammation in rats (Aghlara-Fotovat et al., 2023). In the same study, the hydrogels encapsulating engineered *E*. *coli* strain were retrieved intact from stools. Successful cell encapsulations were reported also using polyacrylamide shells (Tang et al., 2021) and an alginate shell hydrogel methodology (Jeong et al., 2023). The use of environmental strains together with biomass densification through encapsulation might offer a powerful avenue for high-rate toxin removal from the gut and it is therefore important to test removal capacity and virulence of hydrogel encapsulated *T. aminoaromatica* S2 in an animal model to assess p-cresol removal efficacy. It is also key to study if full containment of environmental strains is required to avoid gut colonization or if they would be washed out once released into the gut environment. These questions will determine the next steps for hydrogel engineering requirements.

## Conclusion

A gut-based microbial sink for degradation of p-cresol has high potential for reducing uremic toxicity by avoiding its passage into the blood stream hence slowing kidney disease progression while increasing the patient's quality of life. This study demonstrates a viable proof-of-concept study using hydrogel encapsulated microbes for PBUT removal. We demonstrate that the hydrogel delivery system has shown advantages in biomass densification, cell containment, and showed positive effects on the tolerance to high concentration of p-cresol as possibly present in the gut. Hydrogels may be an efficient and more affordable treatment than dialysis and could act as a personalized needle-free medicine for treating chronic kidney disease patients at home.

## Data Availability

The raw data supporting the conclusions of this article will be made available by the authors, without undue reservation.
