# Supplementary material for: Toward an effective delivery system of a microbial sink of the uremic toxin, p-cresol; an in vitro study with Thauera aminoaromatica S2
Source: Front Microbiol. 2025 May 21;16:1577556. doi: 10.3389/fmicb.2025.1577556 (PMC12136493; doi:10.3389/fmicb.2025.1577556)
Supplement: Supplementary file 1 [file Supplementary_file_1.pdf]

Supplementary Material

Supplementary literature review

Supplementary method

Supplementary results

References

S.1. Supplementary literature review

S.1.1. Degradation pathway of p-cresol by *T. aminoaromatica* S2

Initial reactions of p-cresol degradation by *T. aminoaromatica* S2 were previously reported to involve oxidation of the methyl group to p-hydroxy benzaldehyde and further oxidation to p-hydroxy benzoate (1). The reactions are catalyzed by p-cresol methylhydroxylase and NAD^+ or $NADP^+$-dependent dehydrogenases (2), which were detected in denitrifying organisms (3-5). Denitrifying bacteria further activates p-cresol hydroxybenzoate to p-hydroxybenzoyl-coenzyme A (CoA) by ligase (6, 7) and further to benzoyl-CoA by p-hydroxybenzoyl-CoA reductase (8). According to a review study(2), benzoyl-CoA in denitrifying bacteria is further converted to 3-hydroxypimelyl-CoA and to Acetyl-CoA, possibly through dicarboxylic acid β-oxidation, a glutaryl-CoA dehydrogenase, and a short-chain fatty acid β-oxidation pathway. *T. aminoaromatica* S2 genome shows the genetic evidence of putative enzymes involved in conversions to p-hydroxy benzoate and 3-hydroxypimelyl-CoA (9).

S.2. Supplementary method

S.2.1. Data analysis

Biological triplicates were calculated for arithmetic mean and standard deviations. Cell numbers from the plate counting analysis were based on replicates showing 30 – 300 CFU. The cell quantity was reported as Log CFU mL and the cell encapsulation density was reported as Log CFU (mL hydrogel)$^{-1}$. The p-cresol removal rates by planktonic culture were reported as nmol (Log CFU)$^{-1}$ h^{-1}. The p-cresol removal rates by encapsulated bacteria were reported as nmol (Log CFU)$^{-1}$ h^{-1} or nmol (mL hydrogel)$^{-1}$ h^{-1}. Equations S1 to S6 below showed calculations of p-cresol absolute removal rates for planktonic and encapsulation cultures, stepwise removal rates, and daily bubble tea dose respectively.

$$\frac{\left(p-cresol\, mM\, at\, t\,(end)(mM)\;-\;p-cresol\, mM\, at\, t\,(0)(mM)\right)}{cell\, quantity\,(Log\, CFU)\;\times\;1000\;\times\;incubation\, time,\quad t\,(end)-t\,(0)\,(h)}$$

Equation S1

$$\frac{\left(p-cresol\, mM\, at\, t\,(end)(mM)\;-\;p-cresol\, mM\, at\, t\,(0)(mM)\right)\;\times\;culture\, volume\,(mL)}{hydrogel\, volume\,(mL)\;\times\;1000\;\times\;incubation\, time,\quad t\,(end)-t\,(0)\,(h)}$$

Equation S2

$$\frac{\left(p-cresol\, mM\, at\, t\,(i+1)(mM)\;-\;p-cresol\, mM\, at\, t\,(i)(mM)\right)\;\times\;culture\, volume\,(mL)}{hydrogel\, volume\,(mL)\;\times\;1000\;\times\;incubation\, time,\quad t\,(i+1)-t\,(i)\,(h)}$$

Equation S3

$$\frac{Average\ daily\ presence\ of\ p - cresol\ (mmol\ day - 1)}{removal\ rate\ (mmol\ p - cresol\ mL\ hydrogel - 1\ h - 1)\ \times\ 24}$$

Equation S4

S.3. Supplementary results

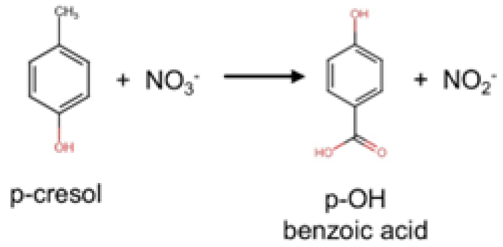

(A)

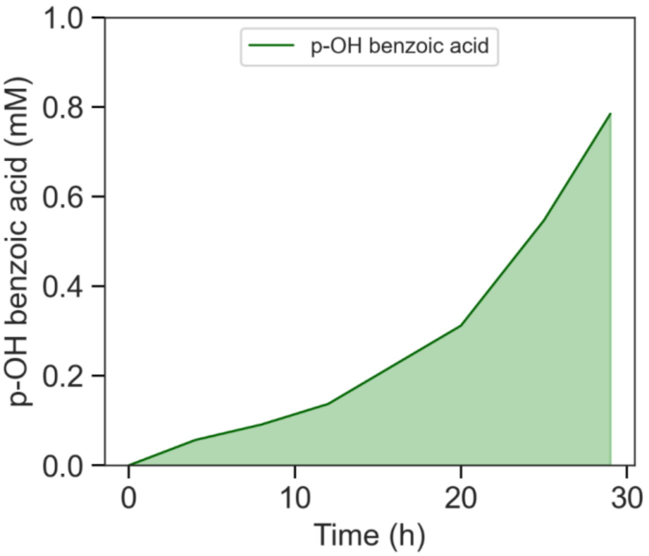

(B)

Figure S1 P-cresol removal by pre-induced planktonic culture of *T. aminoaromatica* S2 at 37 °C. (A) Denitrification of p-cresol by *T. aminoaromatica* S2. (B) p-OH benzoic acid intermediate. The standard deviation is based on biological triplates. Chemical structures were drawn using Marvin JS by Chemaxon.

(A) 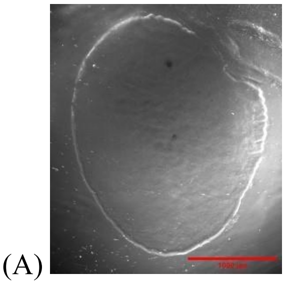 (B) 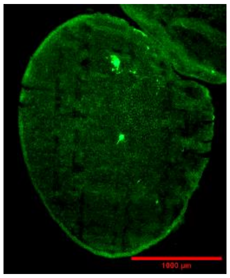 (C) 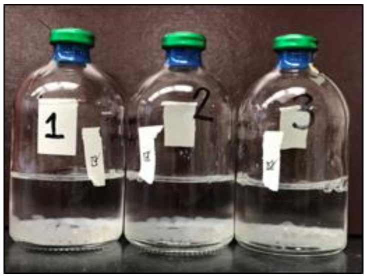

Figure S2 *T. aminoaromatica* S2 hydrogels. A. Cross-sectioned hydrogel (Scale bar of 1000 µm), B. Cells in cross-sectioned hydrogel staining with DNA-staining SYBR fluorescent dyes (Scale bar of 1000 µm), C. Serum bottles for p-cresol removal test containing *T. aminoaromatica* S2 hydrogels and media.

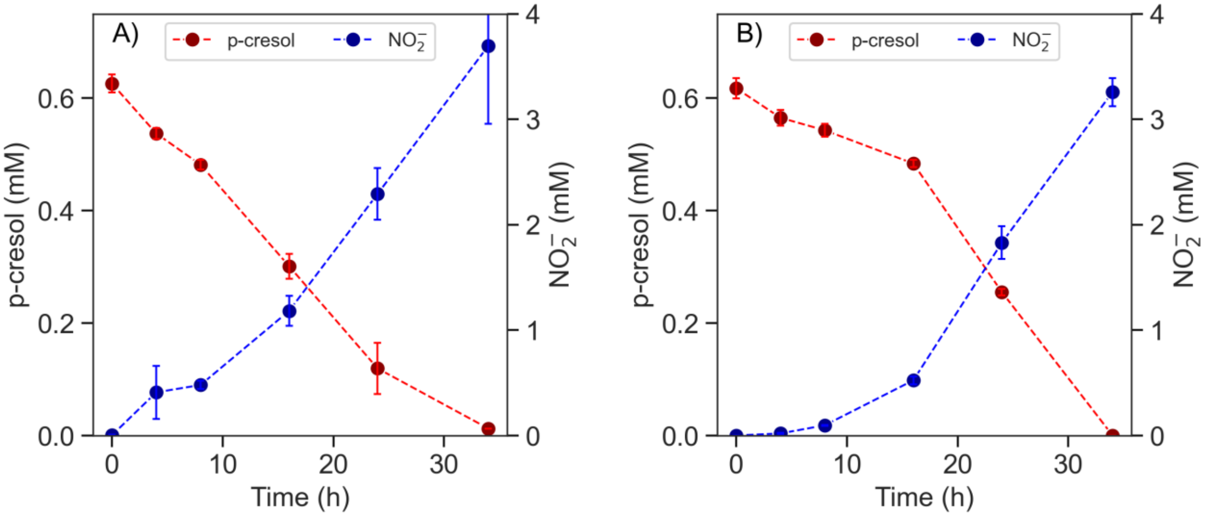

Figure S3 p-cresol removal by encapsulated *T. aminoaromatica* S2 at 37 ºC at pH 7. The p-cresol removal was demonstrated from two different biomass levels, 8.91 Log CFU/mL hydrogel for 1X **(A)** and 9.88 Log CFU/mL hydrogel for 10X **(B).**

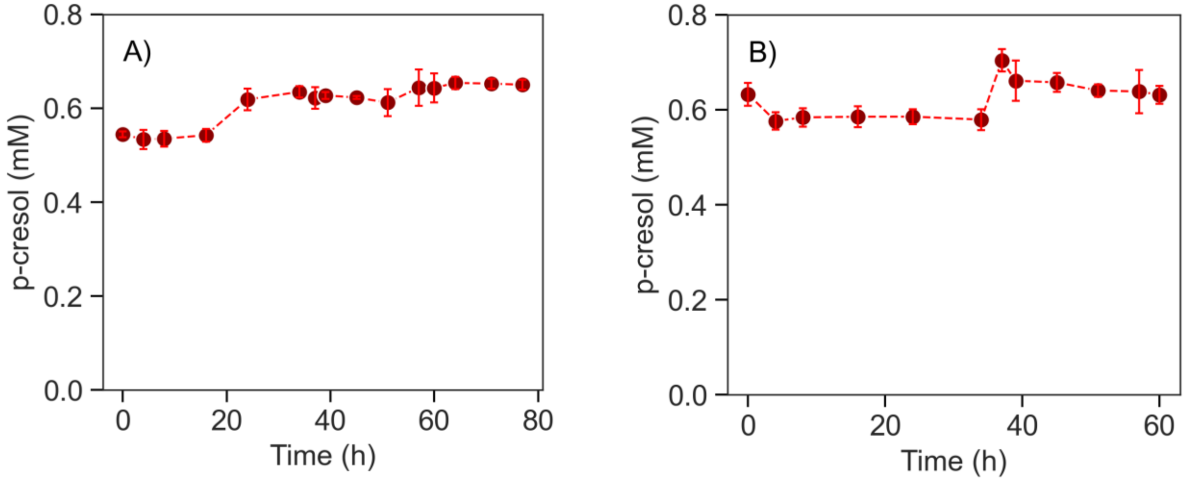

Figure S4 Abiotic control of p-cresol removal test at 37 ºC at pH 7. 1X biomass (A) and 10X biomass (B) of *T. aminoaromatica* S2 was inactivated through 92 °C for 30 min and encapsulated in the hydrogel and tested for p-cresol removal.

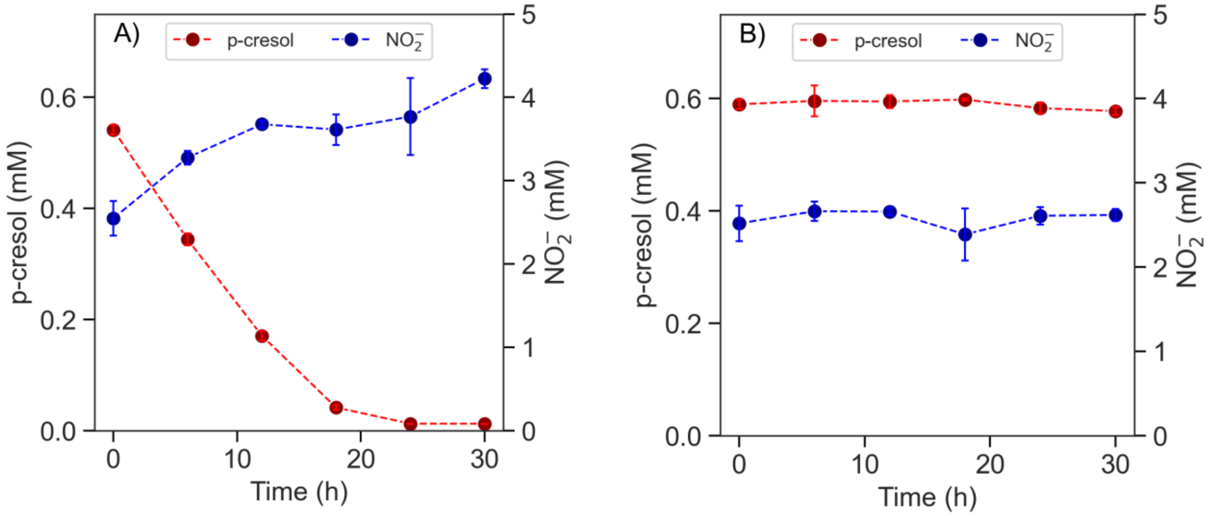

Figure S5 Comparative test to verify encapsulated cell capability in p-cresol removal. The test was a comparison of p-cresol removal between two cultures, (A) the hydrogels in the bulk liquid with cells and (B) the bulk liquid with cells alone.

Table S1 Cell quantification of planktonic and encapsulated cells in this study. In each experiment condition, only the replicate with cell growth was calculated for the cell growth per p-cresol. If replicates were available, the values were reported as arithmetic mean and standard deviation (SD).

Experiment	Condition	Cell number at t_0 (Log CFU)	Cell number at t_{end} (Log CFU)	Removed p-cresol (nmol)	Cell growth, Cell number at t_0 - Cell number at t_{end} (Log CFU)	Cell growth per p-cresol (Log CFU $nmol^{-1}$)
planktonic cell	0.80 mM p-cresol	8.4	8.7	1.9×10^4	0.3	1.6×10^{-5}
	1.6 mM p-cresol	8.1	8.5	2.7×10^4	0.4	1.5×10^{-5}
	2.4 mM p-cresol	8.0	8.8	4.0×10^4	0.9	2.2×10^{-5}
	3.2 mM p-cresol	7.4	6.7	3.9×10^4	-0.6	n/a
	4.0 mM p-cresol	8.3	5.8	4.5×10^4	-2.5	n/a
encapsulated cell with biomass densification	10X biomass	8.0	9.1	5.6×10^4	1.1	1.9×10^{-5}
	1X biomass	7.9	8.6	5.1×10^4	0.7	1.4×10^{-5}
encapsulated cell with p-cresol concentrations	0.80 mM p-cresol	7.5	9.2	3.1×10^4	1.7	5.4×10^{-5}
	1.6 mM p-cresol	8.1 (SD = 0.55)	8.7 (SD = 0.62)	4.1×10^4 (SD = 2.6×10^3)	0.69 (SD = 0.15)	1.7×10^{-5} (SD = 4.9×10^{-6})
	3.2 mM p-cresol	8.5 (SD = 0.45)	7.3 (SD = 0.29)	2.3×10^4 (SD = 1.9×10^3)	-1.2 (SD = 0.24)	n/a

References

1. Seyfried B, Tschech A, Fuchs G. Anaerobic degradation of phenylacetate and 4-hydroxyphenylacetate by denitrifying bacteria. Archives of microbiology. 1991;155(3):249-55.

2. Carmona M, Zamarro MT, Blázquez B, Durante-Rodríguez G, Juárez JF, Valderrama JA, et al. Anaerobic catabolism of aromatic compounds: a genetic and genomic view. Microbiology and Molecular Biology Reviews. 2009;73(1):71-133.

3. Hopper DJ, Bossert I, Rhodes-Roberts M. p-Cresol methylhydroxylase from a denitrifying bacterium involved in anaerobic degradation of p-cresol. Journal of bacteriology. 1991;173(3):1298-301.
4. Rudolphi A, Tschech A, Fuchs G. Anaerobic degradation of cresols by denitrifying bacteria. Archives of microbiology. 1991;155:238-48.
5. Rabus R. Functional genomics of an anaerobic aromatic-degrading denitrifying bacterium, strain EbN1. Applied microbiology and biotechnology. 2005;68:580-7.
6. Biegert T, Altenschmidt U, Eckerskorn C, Fuchs G. Enzymes of anaerobic metabolism of phenolic compounds: 4-Hydroxybenzoate-CoA ligase from a denitrifying Pseudomonas species. European journal of biochemistry. 1993;213(1):555-61.
7. Gibson J, Dispensa M, Fogg GC, Evans DT, Harwood CS. 4-Hydroxybenzoate-coenzyme A ligase from Rhodopseudomonas palustris: purification, gene sequence, and role in anaerobic degradation. Journal of bacteriology. 1994;176(3):634-41.
8. Brackmann R, Fuchs G. Enzymes of anaerobic metabolism of phenolic compounds: 4-Hydroxybenzoyl-CoA reductase (dehydroxylating) from a denitrifying Pseudomonas species. European journal of biochemistry. 1993;213(1):563-71.
9. Kanehisa M, Sato Y, Kawashima M, Furumichi M, Tanabe M. KEGG as a reference resource for gene and protein annotation. Nucleic acids research. 2016;44(D1):D457-D62.
